# Our New York Letter

**Published:** 1886-12

**Authors:** 


### OUR NEW YORK LETTER.

SOME PHASES OF CEREBRAL SYPHILIS DISCUSSED AT THE ACADEMY OF MEDICINE — INSTITUTIONS FOR THE MEDICAL TREATMENT OF THE POOR OF NEW YORK — BASE BALL PLAYING BY MEDICAL STUDENTS, ETC.

*Editors Atlanta Medical and Surgical Journal:*

At a recent meeting of the Academy of Medicine, Dr. Julius Althaus, of London, England, who is a corresponding member of this body, presented a paper entitled "Some Phases of Cerebral Syphilis." The particular object of the paper was to draw attention to some manifestations of cerebral syphilis, with the peculiarities with which we are as yet imperfectly acquainted. He then referred to the two conditions, syphilitic coma and syphilitic hemiplegia. It was not generally known in the profession that syphilis might be the cause of coma coming on quite suddenly in an apparently healthy person, or in one showing symptoms of venereal disease. Syphilis was not generally mentioned in the text-books among the causes of coma, but he considered it of great practical importance, as special treatment was necessary and error in diagnosis might cause fatal results. Dr. Althaus has seen eight cases of this kind in which he considered the diagnosis unmistakable. All the patients gave a history of primary and secondary syphilis and four at the time had specific eruptions. In these cases the coma appeared from eight months to seventeen years after infection. He divided the symptoms into premonitory, initial and final. The premonitory signs were headache, confusion and drowsiness, aphasia, indistinct vision, numbness and paresis. In two cases there were no premonitory symptoms. The initial stage always begins during sleep. Sometimes the patient can be aroused. The eyeballs are retracted into the orbit and diverge somewhat. The pupils are contracted and insensible to light. The muscles of the body are all relaxed, and on lifting a limb there is no resistance and it drops like inanimate matter. Sensation and reflex action are diminished. There is incontinence of urine. The pulse is always slow. Respiration is slow and shallow. The temperature is below the average. This stage lasts from two to five days and is followed either by recovery or the final stage, which leads to death. As recovery is generally due to energetic syphilitic treatment, the doctor may well take to himself the credit of the cure. In the final stage the symptoms of unconsciousness are intensified. Loss of motion and sensation come on. The pulse becomes rapid, the respirations accelerated and the temperature rises. The pupils enlarge. This stage lasts from twenty-four to thirty-six hours and terminates in death. Of the eight cases mentioned, six recovered. Relapses were common. No autopsies were made, but Dr. Althaus thought that the principal lesion in this coma was a gradual occlusion by specific matter of the basilar artery. The prognosis is not very unfavorable if specific treatment be employed from the beginning. The treatment should be partly symptomatic and partly specific. Mercury should be administered either by inunction or the hypodermic method. The writer next spoke of syphilitic hemiplegia. The characteristics of this condition were as follows: Nearly all the patients are males. It only attacks the young. The cases show a peculiar behavior of the deep reflexes. One type was shown by the case of a man, aged twenty-eight, who four years after chancre found his left eyelid drooping. Six months later he had faintness and loss of power gradually stealing over his right side, but the paralysis was incomplete and this partial paralysis was characteristic. Palsies of the nerves of the eye are frequent. The following was given as an instance of the second type: A man, aged thirty-seven, six years after infection, was seized with headache, which lasted six months, when one day he fell down with right hemiplegia and aphasia. Sometime after there followed paralysis of the left leg. In the third type both sides of the body are affected in succession. The last type is that where the paralysis comes on, not more or less suddenly, but quite slowly. There was only one symptom which he considered truly pathognomonic of syphilitic hemiplegia, and this is an excessive exaggeration of the deep reflexes or tendon phenomena, which is wanting in non-syphilitic hemiplegia. Anti-syphilitic treatment was sometimes effectual and sometimes not. He said that in brain syphilis we not only had to deal with specific lesions, but with the consequences of these lesions. A gumma might be absorbed, but when it had already caused wasting of cranial nerves by strangling them, or when the occlusion of an artery led to softening of cerebral tissue, such secondary lesions could not be cured by any amount of mercury or iodide of potassium. It was therefore only possible to cure those in whom the primary had not as yet conducted to secondary lesions. Patients showing the slightest symptoms of specific brain disease should at once be subjected to energetic treatment to prevent the formation of secondary lesions, against which all our remedies were powerless.

### MEDICAL TREATMENT FOR THE POOR OF NEW YORK.

Institutions for the medical treatment of the poor of New York are rapidly increasing in numbers. The managers of the Presbyterian Hospital are erecting a dispensary at Madison avenue and 70th street to cost about $70,000. The building will be one-story high, with a tower, and contain eight rooms. Through the tower, by means of a fan-wheel, the wards of the adjoining hospital will be ventilated. This being a wealthy neighborhood, with three other dispensaries not far distant, there has been considerable opposition expressed by those who think that New York already has too many establishments of this kind. The Lodge and Association Hospital in 8th street has been formally opened. An aid society has recently been organized by representatives of different lodges and societies with the object of raising a fund for the benefit of this hospital, beds being reserved for sick members of those lodges and societies contributing a yearly sum fixed at a minimum of $15.

Plans have been filed for a three-story clinical hospital, to be built at 10th avenue and 60th street, by the College of Physicians and Surgeons. The hospital is to be fire-proof and will cost $130,000. This money was furnished by the late William H. Vanderbilt. There is also a new hospital or home for convalescents in East 16th street, called St. Andrew's Convalescent Hospital, which is under the care of the Sisters of St. John Baptist. Its interior presents handsomely papered walls and frescoed ceilings. Cherry dressing cases are placed at the side of each bed, and chairs and rockers correspond. Handsome mirrors also tend to make the rooms attractive. There are four wards containing twenty beds in all. The hospital is modelled after St. Andrew's at St. Clure, England, and is supported by private contributions. The payment of $10 entitles one to two weeks' stay. The object is to accommodate those whom the over-crowded hospitals are obliged to discharge while yet too ill to return to their daily avocations. The House Staff includes Drs. T. M. Cheeseman, H. D. Nichols and S. F. Morris.

### BASE BALL AMONG MEDICAL STUDENTS.

The game of base ball is becoming popular among the medical students of New York, and two matches have already been played by representative nines of rival colleges. The first was between the College of Physicians and Surgeons and the University Medical College, in which the latter were defeated, the score being eleven to nine. In the second game between the Homœopathic Medical College and the College of Physicians and Surgeons, the latter were beaten by a score of thirteen to five. This result was generally explained on the ground of the homœopaths having a professional pitcher and catcher.

A. F.

*November* 4, 1886.

